# The mammary cellular hierarchy and breast cancer

**DOI:** 10.1007/s00018-014-1674-4

**Published:** 2014-07-31

**Authors:** Samantha R. Oakes, David Gallego-Ortega, Christopher J. Ormandy

**Affiliations:** 1 Cancer Division, The Kinghorn Cancer Centre, Garvan Institute of Medical Research, 384 Victoria St., Darlinghurst, NSW 2010 Australia; 2 St. Vincent’s Clinical School, St. Vincent’s Hospital, University of New South Wales, Darlinghurst, NSW Australia

**Keywords:** Mammary, Breast, Cancer therapy, Cell of origin, Lineage tracing, Tumor heterogeneity

## Abstract

Advances in the study of hematopoietic cell maturation have paved the way to a deeper understanding the stem and progenitor cellular hierarchy in the mammary gland. The mammary epithelium, unlike the hematopoietic cellular hierarchy, sits in a complex niche where communication between epithelial cells and signals from the systemic hormonal milieu, as well as from extra-cellular matrix, influence cell fate decisions and contribute to tissue homeostasis. We review the discovery, definition and regulation of the mammary cellular hierarchy and we describe the development of the concepts that have guided our investigations. We outline recent advances in in vivo lineage tracing that is now challenging many of our assumptions regarding the behavior of mammary stem cells, and we show how understanding these cellular lineages has altered our view of breast cancer.

## Discovery of mammary stem and progenitor cells

Experimental mammary biology leapt ahead with the pioneering work of DeOme and colleagues [1]. They demonstrated that small epithelial fragments of normal or hyperplastic mouse mammary epithelium gave rise to morphologically similar outgrowths when transplanted into de-epithelialized mammary fat pads. Importantly, these outgrowths produced secondary outgrowths when transplanted, confirming that the mammary epithelium contains cells with self-renewing potential, multipotency and cell-autonomous actions, all the characteristics of stem cells [2]. This transplantation technique is one of the most useful methods in mammary biology, used to demonstrate repopulating capacity and self-renewing potential of mammary cells [3, 4], for defining the cell-autonomous role of molecular regulators of cell specification and for isolating their effects from systemic confounders in a variety of applications [5–9]. Using this technique, Smith reported that transplantation of dissociated mammary epithelial cells at limiting dilution revealed three types of outgrowths; lobules, ducts or complete glands. [10]. The ability of serial transplants to produce either lobules or ducts decayed independently of each other, suggesting that this was not just a property of diluted cells [11]. The observations of outgrowths forming only lobules or ducts pointed to the existence of founding cells committed to these fates, but what was not clear was whether these lineage-restricted progenitors cooperated to establish the complete mammary outgrowth, or whether a ‘master mammary stem cell’ could give rise to the entire mammary epithelium [12]. Varmus, Cardiff and colleagues [13, 14] observed that the mouse mammary tumor virus (MMTV) inserts its proviral DNA randomly into the genome of a newly infected pup. This observation provided an ingenious way to track mammary epithelial cells. Exploiting the integration of MMTV to fate-map cells, Kordon and Smith [12] used MMTV to investigate the clonal origins of the mammary gland. These experiments were one of the earliest uses of lineage tracing. CzechII mice were infected with MMTV, and mammary tissue was serially transplanted into uninfected hosts. Subsequent genomic Southern blot analysis revealed identical viral insertion sites in primary and secondary outgrowths. These experiments demonstrated that the mammary epithelium is derived from a multipotent and self-renewing mammary stem cell. Subsequent estimates of the capacity of mammary epithelial stem cells to undergo symmetric cell divisions led to the conclusion that a single mammary stem cell could give rise to an entire functional mammary gland [12]. Similar conclusions were drawn regarding clonally dominant populations in the human breast, where identical X-chromosome inactivation patterns can be observed in contiguous clonal areas of both luminal and myoepithelial cells [15]. Transplantation showed that mammary stem cells were located sporadically throughout the mammary epithelium and concentrated in the cap cells of the terminal end buds. These stem cells were present in the mammary gland at various stages throughout development and were long lived. They also maintained their repopulating capacity throughout the lifespan of a rodent, as they were functionally identical whether isolated from a young or old animal [16], or as transplants or isolated cells from age and parity-matched animals [17]. These experiments established the foundations for our current understanding [18, 19] of the cellular hierarchy of the mammary gland, that a multipotent mammary stem cell can give rise to all the functional-differentiated cells in the mammary gland, that maintenance of tissue homeostasis by localized stem cells produced clonal regions within the mammary epithelial tree, and that progenitor cell populations restricted to lobule or ductal development existed.

## Isolation of mammary stem cell and progenitor cells

First attempts to visualize the mammary stem cell were made by histological investigation, where they were postulated to be morphologically distinct, characterized by pale staining nucleus and cytoplasm containing few organelles (small light cells). These cells were absent in senescent glands, pointing to their regenerative capacity [20, 21], and similar cells with stem cell properties were seen in human tissues when cultured in 3D culture systems [22]. The extensively studied differentiation hierarchy of the hematopoietic system, and the well-developed flow cytometric methods, provided an alternative experimental and organizational paradigm to dissect and describe the cellular hierarchy in the mammary gland [23]. For review, see [24, 25]. Mammary biologists implemented similar strategies combined with intricate tissue dissociation and more gentle antibody-based flow cytometry techniques, to delineate the mammary cellular hierarchy and understand how it is regulated [26]. Some caveats are immediately obvious. Tissues provide positional cues to stem and progenitor cells, the stem cell niche that is absent in the blood. Loss of cellular context during tissue disaggregation, and its random replacement during transplantation, may alter subsequent stem cell behavior. Other caveats are less obvious and more insidious, such as the assumption that the stem cell is an entity rather than an activity, and of a simple, Waddington-like cellular hierarchy, as the paradigm for solid tissues.

Some of the earliest experiments using tissue disaggregation and fluorescence-activated cell sorting (FACS) to isolate different cellular populations in the mammary gland suggested that both the myoepithelial and luminal epithelial fractions contain populations with heterogeneous cellular identities [27, 28]. In 1991, an antibody to epithelial membrane antigen (EMA/25.5/MUC1) was developed that recognized only luminal epithelial cells of the mammary gland throughout development [28]. Its use in conjunction with an antibody against common lymphoblastic leukemia antigen (CALLA/NEP/gp100), which in the mammary gland stained the basally located cells of the myoepithelial cell layer, allowed the mammary epithelium to be fractionated into its basal and luminal components [28]. Later, Dundas and colleagues [27] used the same markers to characterize these two populations in vitro. Curiously, two different basal cytokeratin 14-positive (CK14^+^) clones were derived from the CALLA^+^ fraction, a highly proliferative and predominantly smooth muscle actin negative (SMA^−^) clone and a slowly growing (SMA^+^) clone. The EMA^+^ fraction gave rise to three clones, two slow-growing clones expressing the luminal markers cytokeratin 7/18/19 (CK7^+^, CK18^+^ and CK19^+^) and a third that gave rise to a fast-growing heterogeneous population of basal (CK14^+^), luminal (CK18^+^) cells and bi-lineage (CK14^+^/CK18^+^) cells. All clones could be isolated from both the ductal and alveolar compartments although their proportions differed depending on origin.

The problem with these early studies is that these cell fractions were heavily contaminated with unknown cells derived from the stroma. Smalley and colleagues were aware of this issue and attempted to purify epithelial organoids away from contaminating stromal cells by short-term plating of collagenase-digested tissues [29]. Stromal cells adhered to the plate quickly leaving the unattached organoids in suspension to be isolated and further digested and fractionated by FACS. In this study, luminal cells were isolated using an antibody against mouse milk fat globule membrane antigen (MFGM/33A10) and myoepithelial cells isolated using an antibody to myoepithelial/basal cells (JB6). When cloned in vitro, JB6^+^ basal/myoepithelial cells gave rise to a single slow-growing clone and MFGM-positive luminal epithelial cells gave rise to three different phenotypes, although the myoepithelial (CK14^+^/SMA^+^) and luminal (CK8, CK18 and CK19) identity of these clones was not stable in culture. This issue was solved when each population was cultured in 3D on extracellular matrix (ECM) [30]. Greater than 90 % of all JB6^+^, myoepithelial clones expressed the basal marker CK14 and most were also positive for alpha SMA. Grown on ECM, the MFGM^+^ luminal epithelial cells generated two types of clones, one with flattened morphology and the other rounded that formed complex acinar colonies that invaded the ECM. The flattened clones were characterized by two colony types, a monolayer (Type I, 90 % of flat clones) that expressed both basal and luminal markers (CK14 and CK18) and a multilayered colony (Type II, 10 % of flat clones), which consisted of a CK14^+^/CK18^−^ basal layer intimately in contact with the ECM that surrounded a cuboidal layer with a mixture of luminal CK14^−^/CK18^+^ and bi-lineage cells. The outgrowths that grew out from MFGM, and only in areas where the ECM was thickest, were composed of a central/inner cell mass of luminal CK14^−^/CK18^+^ cells surrounded by an outer layer of double stained bi-lineage and presumably undifferentiated CK14^+^/CK18^+^ cells. Interestingly, luminal-derived clones on ECM were also positive for CK19 and expressed β-casein when grown in lactation medium. These data suggested that the luminal compartment purified using MGFM contains multipotent cells capable of giving rise to both undifferentiated basal cells and functionally competent luminal cells. A human breast progenitor cell separated on the basis of the expression of the MAM-6/sialomucin^+^ (luminal cells) and CALLA^+^ (myoepithelial cells) was discovered using magnetic bead separation [31, 32]. In a specially formulated medium, a small proportion of the MAM-6^+^ cells gave rise to a population of morphologically distinct CALLA^+^, SMA alpha^+^ and vimentin^+^ myoepithelial cells that had lost expression of CK18 and CK19. Hence, it was postulated that all progenitor cell activity resides in the luminal compartment in the human breast [32]. The importance of the luminal origins of this progenitor cell will become apparent below. As significant heterogeneity in morphology and function was still apparent in the myoepithelial and luminal compartments separated by markers mentioned above, the search continued for more specific markers of mammary stem and progenitor cells.

Expression of the ATP-binding cassette family of membrane transporters, notably the expression of Breast Cancer Resistance Protein 1 (Brcp1/Abcg2) is greatest in primitive hematopoietic stem cells and mediates rhodamine 123 and Hoechst 33342 dye efflux [33]. This population is observed as a small negative side population by FACS [34, 35] that can be masked by pre-treatment with voltage-dependent calcium channel blocker Verapamil. Hoechst-33342 dye efflux was explored as a potential marker of multipotent mammary stem cell in mouse and human mammary glands [36, 37]. Side population (SP) cells, which excluded Hoechst 33342, were enriched for stem cell antigen-1 (Sca-1, 80 % of SP [37]), an additional marker of stemness in the hematopoietic system [33, 38]. Transplantation of less than 4,000 SP cells resulted in complete mammary outgrowths containing both ductal and alveolar populations [37], a significant improvement from unsorted mammary epithelium [10], and suggested that the SP was enriched for mammary stem cells [10]. Similar enrichment of undifferentiated and multipotent mammary stem cells was observed for the SP isolated from the human breast [36]. Interestingly, the SP expressed lower levels of cytokeratins and higher levels of vimentin than the non-side population cells and was further enriched for catalytic subunit of telomerase (hTERT) [36], characteristics of primitive stem cells.

Compared to cells enriched from the Hoechst-33342 SP, as few as 1000 Sca-1^+^ cells could give rise to partial or complete mammary outgrowths and suggested that Sca-1 is an improved marker of mammary stem cells. The location of these cells was examined in a Sca-1^+^/GFP reporter mouse. Sca-1 positive cells were found scattered throughout the mammary epithelium, but the greatest number of Sca-1 positive cells were found at the distal tips of extending ducts [37], a localization for stem cells that been suggested before [12]. Interestingly, Sca-1^+^ cells segregated with progesterone receptor (PR) expression, retained label (BrdU) over long periods and possessed an undifferentiated molecular phenotype [37]. These data suggest that the Sca-1^+^ mammary epithelial population is enriched for undifferentiated, long-lived and multipotent mammary stem cells and that Sca-1 appeared to be a better marker of mammary stem cells than dye efflux properties.

Unfortunately, 20 % of the Hoechst-33342 positive population also expressed Sca-1 [37], as did stromal cells [39], hence better markers were needed to enrich for and understand the biology of mammary stem cells. Following on studies by Al Hajj and colleagues that demonstrated the utility of marker for CD24 (Heat stable antigen/HAS/BA-1) in isolating tumorigenic cells from breast cancers (to be discussed in detail below) [40], the Smalley laboratory then investigated the utility of CD24 in fractionating the mammary epithelium [41]. CD45^+^ leukocytes were excluded from dissociated mammary tissue, and the remaining cells sorted into three populations based on CD24 expression: negative (CD24^−^), low (CD24^lo^) and high (CD24^hi^) expression. The CD24^hi^ population almost exclusively expressed the luminal markers CK8/CK18 and had restricted mammary reconstitution potential in limiting dilution transplantation assays. Ninety-four percent of the CD24^lo^ population expressed the basal/myoepithelial marker CK14 and were highly enriched for mammary stem cells. Further fractionation of these compartments came from the addition of an antibody against the glycoprotein Prominin-1 (CD133/AC133), a marker for hematopoietic progenitor cells [42]. Prominin-1 and Sca1 displayed almost identical flow cytometric-staining patterns within the CD24^+^ epithelial compartment, however unlike Sca-1, Prominin-1 was restricted to the CD24^+^ epithelial compartment. Interestingly, expression for Prominin-1 could fractionate CD24^hi^ cells into two compartments; a CD24^+^/Prominin-1^+^ hormone-sensing compartment expressing high levels of estrogen receptor (ER), PR and Prlr and Cited-1; and a CD24^+^/Prominin-1^−^ hormone receptor-negative secretory progenitor cell enriched for in vitro colony-forming ability and capable of milk production [43]. Appropriate steroid hormone receptor patterning and paracrine signaling from hormone-sensing cells was already recognized as a key feature of normal mammary gland establishment and function [44–46]. CD24^lo^ stem cells were predominantly basal cells and did not express the steroid hormone receptors, hence these data demonstrated that most of the stem and progenitor activity in the mammary gland predominantly resides in the steroid receptor-negative basal compartment, and these cells are probably responsive to growth and differentiation signals from the hormone-sensing compartment.

It was clear from these studies that no one marker could be used to purify cell types in the mammary gland. Great advances in the understanding of the breast and mammary cellular hierarchy came from using a combination of different markers to fractionate subsets of the epithelium [3, 4, 47, 48]. Antibodies directed against the luminal antigens EpCAM (ESA) and MUC1 (Mucin-1/EMA) and the basal antigen CALLA were used in combination to fractionate the human breast epithelium and examine their colony-forming ability in in vitro [47]. EpCAM had previously been shown to be a cell surface marker of breast and colorectal carcinomas [49]. Three different colony-forming populations were discovered in these 3D culture systems; the first, a MUC^+^CALLA^−^/EpCAM^+^ population that produced CK8/CK18- and CK19-positive alveolar colonies in ECM; the second, a MUC^−^/CALLA^+^/EpCAM^−^ population that produced colonies of exclusively myoepithelial cells expressing CK14; and lastly, a MUC^− to ±^/CALLA^±^/EpCAM^+^ bi-lineage population that gave rise to large branched colonies of both myoepithelial (CK14^+^) and luminal (CK8/18^+^ and CK19^+^) cells [47]. Hence it was postulated that two progenitor populations maintained cellular homeostasis in the human breast; a bipotent progenitor (MUC^−^ to ^±^/CALLA^±^/EpCAM^+^) that could give rise to both luminal and myoepithelial cells and a luminal (or alveolar) restricted progenitor (MUC^+^/CALLA^−^/EpCAM^+^) that could give rise alveolar colonies. As ESA/EpCAM expression was predominantly found within the luminal layer of human breast tissue, these results suggest that this bipotent progenitor may be luminal in origin. Adding the marker for CD49f (integrin alpha 6/VLA) could further fractionate these human progenitor populations [48]. EpCAM^+^/CD49f^+^/MUC1^+^ sorted cells produced luminal (alveolar) restricted colonies expressing CK8/18 and CK19, akin to the MUC^+^/CALLA^−^/EpCAM^+^ discovered above. The bipotent progenitors were found in a population similarly characterized by EpCAM^+^/CD49f^+^, but had lower expression of MUC, higher expression of CALLA and showed rhodamine 123 efflux properties. Cells derived from this population could give rise to clonal mixed colonies of centrally located EpCAM^+^/CK19^+^ luminal cells surrounded by a halo of CK14^+^ myoepithelial cells and demonstrated bipotent progenitor activity. The expression pattern of CALLA and MUC1 in the EpCAM^+^/CD49f^+^ population suggested this bipotent progenitor might be located in the basal compartment, in contrast to previous findings [47]. The discrepancy between these two studies may reflect the addition of fibroblast feeder layers the later study providing niche-derived signals that maintain the identity of these cells, as had been observed previously in the mouse [30].

Similar results were obtained in the laboratories of Eaves and Petersen. Like the studies above, markers for EpCAM and CD49f were used to fractionate the lineage-negative epithelium into a mammary stem cell population (EpCAM^lo^/CD49f^bright^), lineage-restricted luminal progenitor (EpCAM^+^/CD49f^+^) and mature luminal (EpCAM^+^/CD49f^−^) and myoepithelial lineages (EpCAM^−^ to lo/CD49f^+^) [50, 51]. Using these methodologies, human stromal cells are characterized by an EpCAM- and CD49f-negative immunophenotype and high expression for aldehyde dehydrogenase 1 (ALDH1) [52]. A human breast luminal progenitor can also be identified from the lineage^−^/EpCAM^+^/CD49f^+^ fraction using the combinations of luminal markers CD133 (referred to previously as Prominin 1)/MUC1 and basal makers CD10/THY1 [53]. The basally located MUC1^−^CD133^−^(CD10/THY1)^+^ subset of CD49f^+^ cells contained the majority of the bipotent breast colony-forming cells as assessed by their immune-phenotype in 2D colony-forming assays on collagen-coated plates. The (MUC1/CD133)^+^CD10^−^THY1^−^/CD49f^+^ contained mostly luminally restricted progenitors. Interestingly, progesterone receptor transcripts were enriched in the bipotent progenitor population and not in the luminal restricted progenitors, with the opposite pattern observed for the estrogen receptor, suggesting that the expression of these steroid hormones receptors is not always overlapping. The corresponding populations in the CD49f^−^ population contained mature luminal cells devoid of colony-forming ability. Hence, it was hypothesized that the human breast was composed of undifferentiated bipotent progenitor cells that give rise to committed progenitors that differentiate into myoepithelial and luminal cellular lineages and each cell can by purified based on the expression of EpCAM/CD49f and additional luminal (MUC1/CD133) and basal markers (CD10/THY1).

Parallel populations were obtained using a different combination of cell surface markers in the mouse. In studies from the laboratories of Eaves and Visvader, antibodies for CD24 and the integrins CD49f (integrin alpha 6/VLA) and CD29 (β1 Integrin) were used in combination and examined for their ability to fractionate the epithelium into its cellular compartments. In these studies, mouse lineage cells were depleted using antibodies against CD31^+^ (endothelial), CD45^+^ (hematopoietic), TER119/Ly76^+^ (erythroid) and CD140a/Pdgfr alpha^+^ (fibroblasts, ([4] only) prior to fractionation. CD49f and CD29 had previously been identified as markers of stem cells in the mouse epidermis [54–56]. Given that the mammary gland originates in the ectoderm [57] such as the epidermis, there may indeed be an overlap in the expression of stem and progenitor markers between these organs. Expression of CD24 and high expression of either CD29 or CD49f resolves a population enriched for mammary repopulating units, i.e. those that can give rise to complete mammary outgrowths on transplantation. This population predominantly expressed markers of basal/myoepithelial cells (CK14^+^ and alpha SMA), although the mammary repopulating units/stem cells comprise only a small percentage of this population (1 mammary repopulating unit in 63-64 CD24^+^/CD29^hi^ or CD49f^hi^ cells). Importantly, mammary outgrowths generated from limiting numbers of these mammary repopulating units could be serially passaged, thereby demonstrating their self-renewal capacity. The actual frequency of mammary stem cells may up to 5 % based on the frequency of takes from single cell transplants (6 takes in 102 transplants [3] ); however, the question still remains whether immuno-phenotype of mammary stem cells is indeed that of predominant basal/myoepithelial population. Interestingly, neither Sca-1 expression or Hoeschst 33342 and rhodamine 123 efflux properties were enriched in the CD24^+^/CD29^hi^ or CD49f^hi^ cells, suggesting that the later sorting strategies were superior to these earlier methods. The CD24^+^/CD29^lo^ or CD49f^lo^ population enriched a population of luminal mammary epithelial cells that gave rise to alveolar structures and expressed milk proteins when placed into the 3D culture [3, 4, 58] providing evidence for the existence of a more committed luminal progenitor. Although the efficiency of mammary stem cells to repopulate a mammary gland was significantly reduced at limiting cell dosages [4], both studies provided the first direct evidence that a single mammary stem cell, defined as CD24^+^/CD29^hi^ or CD49f^hi^, could be isolated by flow cytometry and when transplanted could give rise to a functional mammary gland. The existence of this stem cell was demonstrated by Smith and colleagues, and here was its first isolation. In contrast to the mouse mammary epithelium, CD24 expression is confined to the luminal compartment in the human breast, and highlights a critical difference between the two species [53, 59]. Interestingly, mammary cell-autonomous loss of CD24 and CD49f resulted in no defects in mammary reconstitution capacity suggesting that although these two proteins are able to mark and enrich for mammary stem cells, they are not required for mammary stem cell function [60, 61]. Although the markers that define the different populations in the human and mouse vary (Table 1), similarities in the transcriptomes of each population can be observed [62]. For example, the expression of the transcription factors Twist2, Slug, Id4, p63 and Sox11, the Notch ligand Jag2 and WNT/β-catenin pathway members, Fzd8 and Tcf4 are highly expressed in the mammary stem cell populations of both species. In luminal progenitor cells high expression of Elf5, cKIT, CYP24A1 and ALDH1a3 is observed, as is true for Foxa1, Myb, ER, PR, Prlr, Tnfsf11 (Rank ligand) in the mature luminal populations.

**Table 1:** Markers used to identify mammary stem and progenitor cells

Marker	Other Names	Human (H)/mouse (M)	Multipotent stem cell	Luminal lineage	Myoepithelial lineage	Stromal	Mouse mammary phenotype
ALDH1	Aldehyde dehydrogenase 1	H/M	Human breast stem/progenitor cells positive for ALDH1 activity [67], Mouse mammary stem cells CD24^lo^/ALDH high activity [198] Later studies challenge these findings.	Differentiated human luminal cells in human EpCAM^+^/CD49f^+^/ALDH^−^, Undifferentiated human progenitor luminal cells EpCAM^+^/CD49f^+^/ALDH^+^ [66, 68]		High in ALDH1 [52]	
CD10	Common acute lymphoblastic leukemia antigen (CALLA), NEP, gp100	H			Expression [27, 29]		
CD133	Prominin 1, AC133	M		Hormone-sensing cell CD24^high^/prominin^+^, Mature luminal/alveolar cells CD24^high^/prominin 1^−^ [43]			Loss of Prominin1 did not affect mammary stem cell regenerative capacity but resulted in reduced ductal branching and Prlr and matric metalloproteinase-3 expression Mammary proliferation during diestrous (progesterone dependent) was altered [193]
CD14	Lipopolysaccharide binding protein (LBP), LPS-R	M		Alveolar progenitors CD24^+^, CD29^lo^, cKIT^−/lo^/CD14^+^, CD24^+^, Mature alveolar cells CD29lo, cKIT^−/lo^/CD14^−^ [64]			Not formally tested, CD14 knockout mice fertile and were viable [195]
CD24	Heat stable antigen (HSA), BA-1	H/M	CD24^−^ [3], CD24^lo^ [41, 43]	CD24^+^ expression [3, 23] CD24^high^ expression [41, 43] CD24^+^ human luminal lineage [59]	CD24 high expression [41, 43]	CD24 negative [41, 43, 62]	CD24 KO mammary stem cells showed no defect in mammary repopulating capacity. CD24 KO mice had increased ductal branching [60]
CD29	β1-Integrin	M	Mouse stem cell-enriched CD24^+^/CD29^hi^ [3]				Blocking Integrin β1 reduced ductal branching and alveolar bud development [61] Integrin β1 conditional KO showed decreased alveolar differentiation and Integrin β1 KO stem cells had reduced repopulating capacity [120]
CD44	H-CAM, Pgp-1	H	Tumor-initiating cell CD44^+^/CD24^−^/low [40]				Not formally tested, CD44 KO mice were viable [196]
CD49b	Integrin alpha 2, VLA-2	M		Colony-forming progenitors EpCAM^+^/CD49f^lo^/CD49b^+^ [66]			
CD49f	Integrin alpha 6, VLA-6	H/M	Mouse stem cell-enriched CD24^+^/CD49f^hi^ [23]	Human luminal lineage CD49f^neg^/EpCAM^+^ [52]	High CD49f marks human basal cells [52]	Human stromal cells CD49f^−^/EpCAM^−^ [52]	No mammary phenotype [61]
CD61	Integrin beta 3, GPIIIa	M		Mouse luminal progenitor CD24^+^/CD29^lo^/CD61^+^, Mouse mature luminal CD24^+^/CD29^lo^/CD61^−^ [5]			
cKIT	CD117/KIT	H/M		Luminal progenitor cKIT high [52, 62]. CD24^+^ CD29^lo^ cKIT^+^ [64]			
Dye Exclusion: Hoechst Dye 33342 or Rhodamine 123		H/M	Exclusion [36]				
EMA/MUC1	Epithelial membrane antigen/25.5	H/M		Expression [28]			
EpCAM	ESA (epithelial surface antigen, epithelial cell adhesion molecule, Ly74, CD326	H/M	Human stem cell-enriched CD49f^+^ EpCAM^hi^ [51], EpCAM^lo^/CD49f^bright^ [50]	Luminal lineage EpCAM^hi^ [51, 52] CD49f^−^/EpCAM^+^ [52]	CD49f^+^/EpCAM^−/lo^ human myoepithelial lineage [50, 51]	Human stromal cells CD49f^−^/EpCAM^−^ [52]	Not formally tested, EpCAM knockout mice embryonic lethal [194]
JB6	Basal/myoepithelial marker	M			Expression [29]		
MFGM (33A10)	Milk fat globule membrane protein/luminal marker	M		Expression [29]			
Sca1	Stem cell antigen	H/M	Sca1^+^ [37]	CD24high/Sca1^+^ [43]	CD24^lo^ [43]	CD24negative/Sca1^+^ marks a non-epithelial population [43]	
Thy1/CD90		H	Human Bipotent progenitor EpCAM^+^/CD49^+^ MUC1^−^/AC133^−^ (CD10/Thy1)^+^ [53]	Human luminal restricted progenitor EpCAM^+^/CD49^+^ MUC1^−^/AC133^−^ (CD10/Thy1)^+^ [53]	MUC1^−^/AC133^−^ (CD10/Thy1)^+^ [53]		Not formally tested. Thy1 KO fertile and were viable [197]

Table outlines the common markers (marker) and their other names (Other Names) used to isolate mammary stem and progenitor cells in mouse (M) or human (H) tissues. The utility of each marker to enrich for populations of multipotent stem cells, cells from the luminal and myoepithelial lineages and stromal cells is outlined and references for each study indicated. The phenotypic consequence of ablation of each marker in mice and relevant references are outlined where applicable

To further characterize the luminal populations, the Visvader laboratory used an antibody for CD61 (Integrin beta 3/GPIIIa), which defines the megakaryocytic cell lineage. CD61 expression defines two overlapping populations in the CD24^+^/CD29^lo^ luminal subset in the mouse [5, 63]. This population is highly enriched for luminal (CK18^+^) colony-forming cells that give rise to alveolar structures in 3D culture, whereas the CD61^−^ population had luminal morphology, expressed luminal markers (CK18) and was devoid of colony-forming ability. The proportion of CD61^+^ positive cells dramatically decreased during pregnancy (decreasing 15-fold by 18 days post coitus) consistent with increasing differentiation toward mature alveolar cells characterized by a CD24^+^/CD29^lo^/CD61^−^ immuno-phenotype [5]. Hence, CD61 expression in the CD24^+^/CD29^lo^ defined a luminal progenitor (CD61^+^) and mature luminal cells (CD61^−^). Later, the Visvader laboratory demonstrated that the CD24^+^/CD29^lo^ population could be alternately fractionated into a cKIT^+^ progenitor population that likely overlaps with the previously identified CD24^+^/CD29^lo^/CD61^+^ luminal progenitor population [64]. cKIT/CD117 or receptor tyrosine kinase is highly enriched in the luminal progenitors of both human and mouse epithelium [62]. Furthermore, negative or low expression of cKIT in the CD24^+^/CD29^lo^ population that also expressed CD14 (cKIT^−/lo^/CD14^+^) can further purify the luminal fraction into an alveolar progenitor population that expressed milk in the absence of a lactogenic stimulus, and a mature luminal population (cKIT^−^/CD14^−^). More recently, the activity of the MMTV promoter within mammary epithelial cell populations was reported to distinguish a multipotent progenitor population from within CD24^+^/CD29^lo^ cells with alveoli-forming capacity, particularly in response to pregnancy, but without potential for self-renewal by serial transplantation [65]. These progenitors showed an ELF5 high phenotype, while the non-alveoli forming progenitors showed a pattern of gene expression indicative of the hormone-sensing lineage. Multipotency was inferred from the formation of alveoli and milk protein production, but also that p63^+^ myoepithelial cells were formed. Whether these cells are able to make both myoepithelial and epithelial cells remains controversial. If so, this may represent a new lineage of alveolar-myoepithelial cells, in contrast to a separate ductal myoepithelial cell lineage, which could also explain the contrasting myoepithelial cell susceptibility to apoptosis during involution, and their net-like, rather than sheath-like, arrangement in alveoli and ducts, respectively.

The currently definitive work on progenitor cell phenotypes was published in 2012 by Shehata, Stingl and colleagues [66]. Having distinguished luminal and basal populations in Lin^−^ cells from mouse mammary gland using EpCAM and CD49f, they divided the luminal population into three subpopulations on the basis of Sca1 and CD49b staining. High colony-forming frequency and low multipotent stem cell activity were associated with high CD49b expression, indicating this population comprised progenitor cells with either some limited capacity for dedifferentiation to stem cells, or with some incomplete stem cell differentiation. High estrogen receptor expression (ER) and expression of GATA3 and FoxA1 were found in progenitors marked by high Sca1 staining, while low/no ER expression but high Elf5 and Lmo4 expression, along with expression of milk proteins such as Mfge8 and Lalba, basal markers such as K5 and high ALDH activity were found in the progenitor population with low Sca1 staining. High ALDH activity has previously been shown to mark human mammary stem cells [67] and here marks a ER^+^ luminal progenitor cell. Similarly, high ALDH activity together with high expression of ERBB3 can purify luminal alveolar progenitor cells from the EpCAM^+^/CD49f^+^ luminal progenitor compartment isolated from human tissue [66]. These results are supported by additional work in human tissue showing that ALDH activity is in fact the lowest in EpCAM^−/lo^/CD49f^+^ mammary stem and bi-lineage progenitors but is greatest in the EpCAM^+^/CD49f^+^ luminal progenitors, a point when mammary cells become committed to the luminal lineage [68]. Thus, a clear progenitor cell dichotomy exists with gene expression patterns indicative of a determined cell fate, either as the founders of the lineage acting as hormone-sensing cells in the case of the ER/FoxA1/Gata3 progenitors (also called luminal or ductal progenitors) or as founders of the milk-secreting cell lineage in the case of the Elf5/Lalba/Mfge8 cells (also called alveolar progenitors).

## Lineage tracing to map cell fate decisions

The various markers discussed above enrich, but do not purify, mammary stem and progenitors cell populations. It is unclear how tissue disaggregation, niche removal and its random replacement during transplantation alter stem and progenitor cell phenotype and activity [19, 69, 70]. A potential solution to these issues is to genetically mark the stem and progenitor cells, and then chase the mark throughout development, to define the cell lineage that the target cells produce. The key to lineage tracing is the initial accurate marking of the intended starting population, especially when targeting populations other than multipotent stem cells, to avoid the confusion caused by the marking of off-target cells. These techniques, coupled to high-resolution imaging have recently become a reality [69, 71, 72] and the pioneers of these techniques have discovered both the advantages and pitfalls of these techniques. The gastric epithelium is a prime example [71, 73–75]. These studies employed the Lgr5 promoter, identified as a putative stem cell marker, to drive the marking allele, a Tamoxifen-inducible Cre that activated a LoxP-flanked Rosa26LacZ reporter. Lgr5^+^ cells were restricted to the base of the pylorus crypts, and lineage tracing revealed that they were rapidly self-renewing cells that gave rise to multiple gastric epithelial progeny throughout adulthood, hence possessing all the qualities of a stem cell [73]. When these cells were deleted, however, there was no effect and the crypts remained intact. This revealed the presence of a second stem cell pool, marked by Bmi-1 expression and label retention and located at the +4 position, that produced the Lrg5^+^ cells but which could also directly produce the cells of the crypt. Therefore, there are two types of stem cell in the intestine, and whether one or both are stem cells remains controversial. A worrying aspect of these experiments is that the tamoxifen used to mark the Lrg5^+^ cells caused the apoptotic death of these cells [76, 77], which enhanced the production of cell progeny from Lrg5^+^ cells. Apoptotic suppression by genetic overexpression of Bcl-2 or knockout of Chk2 caused enhanced production of Bmi1 cells, pointing to an apoptotic artifact, but using a background, that may have influenced the observations made. A further lesson from the studies of the intestinal crypt is the ability of cells to dedifferentiate to become stem cells. Buczaki and colleagues [78] used a version of label retention, where the CreA fragment of the CRE recombinase was fused with Histone 2B. Induction (using beta-naphthoflavone) in double transgenic mice that express CreB from the Rosa26 locus and the histone 2B-CreA fusion gene, caused the CreA fragment to be retained on histones of cells that had not divided. Administration of intravenous dimerizing agent resulted in heterodimerization of CreA and CreB to form a functional recombinase, which marked non-proliferative cells. These cells were chased and shown to become differentiated Paneth cells, but could revert to become stem cells following tissue damage that repopulated the crypt. Thus, the definition of a stem cell needs to be carefully considered. In solid tissues, stemness may be an activity, rather than an entity, that is available to many cells to meet the spatial constraints of solid tissue regeneration. This may be a key difference to the stem cells of the immune system, which have provided the paradigm for the mammary stem cell hierarchy. As we will see, the problems of tamoxifen deletion of stem cells and injury-induced formation of stem cells have most recently arisen in the interpretation of mammary lineage tracing.

As discussed above, the earliest examples of lineage tracing in the mammary gland were performed in the Smith laboratory using MMTV integration [12], and the same laboratory was later involved with the description of an adjunct mammary epithelial cell population [79] that was later termed “parity-induced” mammary epithelial cells (PI-MECs) [80], but is actually better described as “parity-labeled” or “parity-amplified” as they are present before pregnancy [81]. The description of this population pioneered inducible lineage tracing in the mammary gland [79]. Here, transgenic mice-expressing Cre recombinase under control of the mammary (and pregnancy)-specific promoter for whey acidic protein (WAP) was crossed with mice ubiquitously expressing a floxed Rosa/LoxP/Stop/LoxP/lacZ gene. Lineage marking was induced by pregnancy. Cre-mediated recombination resulted in the permanent expression of β-galactosidase, detected as a blue color after X-Gal staining. Not all marked alveolar cells underwent apoptosis during involution, and up to 20 % of cells remained marked in involuted parous glands, compared to 1 % in nulliparous glands. Transplantation showed that these cells could produce both ducts and alveoli. These cells accumulated as the lactation-deficient phenotype in Prlr heterozygote animals resolved with repeated pregnancy and they serve as the cell of origin for neu-induced tumors [80]. Interestingly, although the vast majority of these cells are restricted to the alveolar lineages, some cells show multipotency, seen as myoepithelial cells staining for both beta-galactosidase and smooth muscle actin [82], or using a WAP-driven Cre-activated GFP reporter, as GFP^+^ cells staining for K14. GFP^+^ cells were able to reproduce entire mammary glands in 4 of 11 attempts following the transplant of 10^4^ cells, suggesting multipotency [83]. GFP^+^ cells were observed within the CD24^+^/CD49f^hi^ population.

WAP is thought to be a milk protein and so is considered to mark differentiated cells, however, WAP is a direct transcriptional target of the ETS transcription factor Elf5 [84]. Thus, WAP-driven lineage tracing may be equivalent to Elf5-driven lineage tracing, albeit at reduced efficiency. We have hypothesized that Elf5 expression has two major effects in the mammary gland [85, 86]. When it is first expressed it causes stem cells to become alveolar progenitor cells [87, 88], therefore defining the progenitor cell populations by inducing epithelial characteristics [89]. Further increases in Elf5 expression, in response to hormonal influences [88] force a fate decision within the progenitors, to become the Elf5^hi^ secretory population, or if estrogen signaling dominates to become the Elf5^lo^ ER^+^ hormone-sensing population. WAP-directed lineage tracing has the potential to infrequently label some very early progenitor cells, which may still possess multipotency, and some ER^+^ sensor cells, but most of the labeled cells will be the progenitors of secretory cells and their mature progeny that form the alveoli. Thus, the observations of PI-MECs with multipotency, self-renewal and common long-lived alveolar progenitor activity can be readily understood. The relationship of PI-MECs to alveolar progenitors, the sensor cell population and Elf5 expression has recently been defined by the laboratory of Pietersen and colleagues [90]. Using the WAP–CRE approach to activate a floxed-stop-Rosa26 YFP cassette they observed overwhelming labeling of the ER^−^ secretory alveolar lineage, with just 6 % of the ER^+^ hormone-sensing lineage showing labeling. As no YFP^+^ cells were observed in the basal compartment by flow cytometry, or by immunofluorescence or co-staining with SMA, Pietersen and colleagues speculate that transplantation may be the cause of observations of PI-MECs contributing to the myoepithelial lineage, though in these types of experiments labeling inefficiency may also be a cause. Conclusions based on the failure to make an observation must be made with caution. qPCR measurement of Elf5 in flow-sorted populations from involuted parous glands showed that alveolar progenitors expressed Elf5 and that a subset of these progenitors were labeled with YFP. The equivalent progenitor activity of labeled and unlabeled progenitor populations was seen in the YFP expression pattern during subsequent pregnancies, with alveoli showing either near complete or no/low frequency of labeling, indicating their clonal origin and functional equivalence [90]. These results support the contention that WAP-driven labeling reports Elf5 activity, though at well less than 100 % efficiency, and marks long-lived alveolar progenitors. Elf5-driven fate mapping was reported this year using doxycycline-inducible activation of the confetti reporter under the control of the proximal region of the Elf5 promoter [91]. Only luminal cells were marked. These cells were long lived and a number contributed to the formation of the alveoli during pregnancy, seen as multicolored alveoli. Again the conclusions that were drawn regarding the absence of labeling in the ducts and of myoepithelial cells must be treated with caution given that marking cannot be 100 % efficient.

Blanpain and colleagues [92] investigated the issue of transplantation altering stem cell activity. They observed that K14-driven and doxycycline-inducible marking of fetal mammary cells during late pregnancy labeled both epithelial and myoepithelial cells, but that the same procedure immediately postpartum, or during puberty or pregnancy, only labeled myoepithelial cells. A K5-driven and tamoxifen-inducible system reproduced this result [92]. Using luminal drivers such as K8 and K18, and tamoxifen induction, only labeled epithelial cells were observed. When K14-driven and doxycycline-induced labeling of cells was performed in a 4-week-old mice, then enzymatically disaggregated and transplanted, the myoepithelial restriction was broken and both luminal and myoepithelial cells were labeled. Recombination transplantation experiments using these marked myoepithelial cells mixed with increasing ratios of unmarked luminal epithelial cells showed that the presence of luminal epithelial cells inhibited the multipotency of the myoepithelial stem cells. Thus, the conclusion drawn was that the epithelial and myoepithelial lineages share a common stem cell in utero, but from birth the gland is maintained by unipotent stem cells that are restricted to the epithelial and myoepithelial lineages. Postpartum multipotent stem cell activity is either revealed, or produced, by transplantation. Physiologically, the multipotent stem cell may be very important for recovery from injury, such as mastitis, which has a very high incidence in mammals during lactation. Parallels between these unipotent stem cells (or are they better called progenitor cells?) can be drawn with the luminal- and myoepithelial-derived progenitors discovered in earlier studies using markers of luminal cells (MFGM/MAM-6/EpCAM) and myoepithelial cells (JB6/CALLA/CK14), respectively [27, 28, 31, 32]. Whether these progenitors represent the same cellular populations remains to be determined.

Since the publication of this work by Blanpain and colleagues, a number of papers have supported or questioned its conclusions. van Amerongen and colleagues [93] used Axin2-driven and tamoxifen-inducible lineage tracing of Wnt-responsive cells. When mammary cells were labeled during the fetal period, marked cells were restricted to the luminal fate with no marked basal cells observed by flow cytometry. Labeling postpartum produced the opposite result, labeling of basal not luminal cells. When these cells were transplanted both luminal and basal layers of the ductal tree were produced, and alveoli were now derived from cells previously marked as basal. This pattern of marking was transplantable and suggests activation of multipotent stem cell activity by transplantation. Post-pubertal animals continued to show a basal pattern of labeling. During pregnancy this pattern persisted, but newly formed alveoli showed labeled cells that produced milk at the end of pregnancy. After multiple pregnancies and involutions, this labeling pattern persisted in the ducts and regressing alveoli. These findings, which are compatible with the Blanpain model, show that Wnt activity occurs in both the basal and luminal progenitor compartments, but whether one becomes the other, as would occur in a multipotent stem cell model, will require labeling with confetti [94] or brainbow [72] to demonstrate clear clonal decent. The ability of the mammary fat pad to influence a mammary-like differentiation fate is well known [95, 96], so there is precedent for mammary cells being so influenced.

Visvader, Lindeman and colleagues [91] have used K5-driven doxycycline-inducible linage tracing to demonstrate that multipotent stem cells do operate during normal development and are not simply a product of transplantation. The use of confetti mice allows the stochastic production of multiple colors, allowing the simultaneous observation of multiple clones within the same region of mammary architecture [94]. Using this model in adult mice, they observed labeled cells in both luminal and myoepithelial lineages, and these cells contributed to alveolar morphogenesis. A K14-driven and tamoxifen-activated model, and a Lrg5-driven tamoxifen-activated model produced the same result-labeled cells in both myoepithelial, luminal and alveolar cells, consistent with the activity of a multipotent stem cell during normal mammary development. The disparity between this study and the previous studies is stark. Possible reasons include model differences in promoter specificity and/or transcriptional activity due to transgene insertion sites or the different promoter fragments utilized, producing labeling in different cell lineages and with different efficiencies. The use of 2D rather than 3D confocal imaging is another difference. The ability of high doses of tamoxifen to kill mammary stem cells offers another distinction; however, doxycycline is not thought to cause this problem (as yet), and many of the divergent results were produced using doxycycline, not tamoxifen to induce labeling. Drawing on the lessons learned from the mapping of the cellular hierarchy of the intestinal epithelium, where fast and slow stem cells operate, and phenotypic plasticity occurs, the resolution of these disparate observations is likely to be in the undiscovered complexities of the mammary hierarchy.

Lgr5^+^, a marker of pyloric stem cells, has also been used to trace mammary cell lineages [97]. Just 5 % of the CD24^mid^/CD49^hi^ mammary stem cell-enriched population expresses Lgr5. Interestingly, lineage tracing of Lgr5^+^ cells from the first day after birth resulted in labeling of the luminal progenitor cell compartment only, and only when cells were traced from the twelfth day of birth did Lgr5^+^ cells label the myoepithelial compartment. The switching in the Lgr5^+^ progenitor cell activity may reflect changes in Wnt signals from the microenvironment at different developmental stages. Importantly, these results add further evidence that distinct mammary progenitors have discrete roles at various stages of development.

There may be several new progenitor cells that are yet to be revealed. Fascinating evidence for this comes from the study of Sale and colleagues who traced Notch2 expression [98]. Small Notch2-expressing cells formed rings around a large Notch2 cell, and these colonies occurred in a repetitive and equally spaced arrangement within the terminal end bud and the trailing duct, where the pattern persisted post-pubertally. Large Notch2^+^ cells appeared with random frequency throughout the duct. All Notch2^+^ cells expressed keratin 8 and not keratin 14 showing them to be exclusively epithelial. They fall within the CD24^+^CD29^low^ population and many are also CD61^+^ suggesting there are potential progenitor cells among them. Transplantation showed no outgrowths from these populations and lineage tracing showed no clonal alveolar development, indicating that these cells are not classical progenitors. Small cell strings were localized at tertiary branch points, and forcing the expression of the diphtheria toxin receptor under Notch2 control resulted in the cessation of branching when diphtheria toxin was administered mid-puberty. At pregnancy, diphtheria toxin prevented correct alveolar morphogenesis, resulting in poorly organized hyperplastic regions where alveoli should have formed, and little milk protein synthesis. These cells show morphologic similarity to the small light cells and large light cells previously identified using ultra-structural microscopy in the Smith laboratory [20]. The source of these cells remains unknown. Regardless, it is clear that the lineages within the mammary gland are far more complex than currently realized.

Lineage tracing studies have therefore identified new progenitor subtypes that have discrete roles in a stage-dependent context. Importantly, these techniques have revealed key differences in the lineage potential of mammary stem and progenitors in unperturbed glands in vivo compared to those where these populations have been isolated by flow cytometry and examined in mammary reconstitution assays. The extent of the overlap between these new progenitor subtypes and those identified in early morphological and prospective isolation studies remains to be determined. The advent of superior lineage tracing techniques, for example those that employ Brainbow or Confetti transgenic mice [72, 94], paves the way for a more advanced understanding of the mammary cellular hierarchy. The challenge now is choosing appropriate genes to drive lineage marking. The best genes for this purpose are those that define the various lineages within the mammary gland.

## Regulation of the mammary cellular hierarchy

The Smalley laboratory showed that a hormone-sensing population exists separately from stem cell activity [43]. It is widely accepted that steroid receptor expression segregates from proliferating cells, and that paracrine signals from steroid receptor-positive cells must contribute to the proliferation of adjacent hormone receptor-negative cells [44, 45, 99]. The mammary stem cell-enriched population is likely negative for the expression of steroid hormone receptors, so the question remains, how do steroid hormones regulate the proliferation and self-renewal abilities of mammary stem cells? That steroids do influence stem cells was demonstrated by the Khokha and Visvader laboratories [6, 100]. At diestrous, when progesterone levels are highest, the absolute numbers of CD24^+^/CD49f^hi^ mammary repopulating units in the mammary gland increase by up to 14-fold over that observed at estrous [100]. Furthermore, the size and repopulating capacity of the CD24^+^/CD29^hi^ mammary stem cell population decreased in ovariectomised mice and increased in mice treated with estrogen [6] and progesterone [6, 100]. Three weeks of treatment with the aromatase inhibitor Letrozole, which inhibits the synthesis of estrogen, is sufficient to significantly decrease the numbers and activity of mammary stem cells [6]. These results suggested that a paracrine mediator is responsible for the proliferation and maintenance of mammary stem cells, and a likely candidate was the progesterone-regulated RankL [100]. In fact, it is now understood that PR has two modes of mitogenic action in the mammary gland; firstly a cyclin D1-dependent stimulation of proliferation of PR^+^ cells, and secondly a RankL-mediated paracrine action on nearby PR^−^ mammary epithelial cells [101]. Indeed, treatment of mice with a RankL-neutralizing antibody decreased both the numbers and the colony-forming ability of CD24^+^/CD29^hi^ mammary stem cell-enriched population [6]. Hence, mammary stem cell maintenance likely occurs via paracrine signals that include RankL, which is released from neighboring steroid receptor-positive cells where it is induced by steroid hormones. RankL activities are not confined to the mammary stem cell compartment as RankL can also stimulate the expression of the transcription factor Elf5 in progesterone receptor-negative luminal progenitor cells and promote alveolar development [88]. In these experiments, progesterone treatment of mice alone resulted in an expansion of the CD24^+^/CD29^hi^ mammary stem cell-enriched compartment, confirming progesterone driven action on mammary stem cells. Progesterone also resulted in a depletion of the CD24^+^/CD29^lo^/CD61^+^ luminal progenitor cell population and drove its differentiation toward the CD24^+^/CD29^lo^/CD61^−^ mature luminal cell. Forced expression of Elf5 in the luminal compartment, together with progesterone treatment, completely depleted the luminal progenitor population and resulted in the expansion of the mature alveolar lineage. RankL-neutralizing antibody blocked the action of progesterone in this model and suggested that most of the action of progesterone on luminal cell differentiation is dependent on RankL paracrine signaling. Forced Elf5 expression also reduced stem cell numbers, suggesting an action of Elf5 to differentiate these cells. Hence, both mammary stem and luminal cells are regulated by paracrine signals from committed steroid receptor-positive luminal cells.

Paracrine signals from basal epithelial populations may also control the proliferation and fate of the luminal epithelium. Forster and colleagues provide evidence for this. Genetic deletion of the transcription factor p63 in K14-positive basal epithelium resulted in profound defects luminal cell proliferation and differentiation during pregnancy [102]. No changes in basal cell number or proportion were detected; however, the proportion of luminal progenitors (CD24^+^/CD29^lo^/CD61^+^) accumulated in the mammary glands of mice, suggesting that they had failed to differentiate into mature alveolar cells. These p63-competent luminal epithelial cells could be made to differentiate into large alveolar-like colonies when cultured in complete medium suggesting that a p63-dependent paracrine factor released from basal cells may be responsible for their fate. This factor was found to be neuregulin (Nrg1, heregulin-alpha), produced by the basal epithelia, and found to be a direct transcriptional target of p63. Exogenous Nrg1 rescued the lactation phenotype in recombined glands with basal cell p63 deletion, and this occurred through activation of the ERBB4/Stat5A in neighboring luminal epithelial cells [102]. Hence, in addition to paracrine signals that travel from luminal sensor population to other luminal cells and basally located mammary stem cells, basal cells themselves can send signals back to regulate the fate of the luminal epithelium. This ‘teamwork’ likely underlies every aspect of breast development and homeostasis and is only beginning to be unraveled [103].

Transcription factors govern large genomic networks and regulate cell fate specification within the mammary cellular hierarchy. For example, the expression of the transcription factor Gata3 is highly enriched in the CD24^+^/CD29^lo^/CD61^−^ mature populations and mammary specific loss of this transcription factor resulted in at least a twofold increase in the size of this population, whether isolated from a virgin gland or during pregnancy. Furthermore, forced expression of Gata3 in the CD24^+^/CD29^hi^ mammary stem cell-enriched population resulted in the expression of markers of luminal cell differentiation and milk proteins. These results suggested that Gata3 is essential for luminal cell fate specification at multiple stages of the mammary cellular hierarchy [5]. The fate of the CD24^+^/CD29^lo^/CD61^+^ luminal progenitor is also driven by the Ets transcription factor Elf5. Mammary specific loss of Elf5 results in complete failure of alveolar morphogenesis and milk secretion [8]. Conversely, forced expression of mammary specific Elf5 results in precocious alveolar differentiation of virgin epithelium and milk production together suggesting that Elf5 is the master regulator of alveolar morphogenesis. A greater proportion of the CD24^+^/CD29^lo^/CD61^+^ luminal progenitor population is observed in Elf5 knockout mammary glands and the converse was true in transgenic animals expressing Elf5, where forced expression of Elf5 eroded the CD24^+^/CD29^lo^/CD61^+^ luminal progenitor and specified its differentiation along the alveolar cell lineage. It is likely that Elf5 expression is a key determinant in the differentiation of stem cells to progenitor cells, via its epithelializing influence [86, 87], which distinguishes the myoepithelial and epithelial lineages and correlates with the loss of epigenetic silencing of Elf5 in epithelial cells, but not the stem and myoepithelial compartments. Elf5 is then also involved in the determination of the two progenitor lineages via a mutual negative regulatory loop involving ER and FoxA1, so that one or the other phenotype is fixed by dominant transcriptional repression of the other [85, 86, 104].

The NOTCH signaling pathway has also been shown to play a role in lineage specification in the mammary gland. The Notch pathway involves juxtacrine signaling between cells in direct contact with each other via the Notch transmembrane ligands (Delta-like DLL1, 3, 4 and Jagged Jag1–2) of one cell and the Notch transmembrane receptors (Notch 1–4) on a neighboring cell [105]. The cleaved intracellular domains of the Notch receptors (NICD) translocate to the nucleus where they also activate programs of transcription. The Notch active intracellular domain is released by gamma secretase cleavage permitting its translocation to the nucleus where it interacts with the CSL complex to activate the transcription of target genes that include Hes and Hey. The expression of Notch ligands, receptors and transcriptional targets is differentially regulated in mammary stem, luminal progenitor and mature luminal enriched populations [106]. Interestingly, Notch4 was first discovered as an integration site for MMTV, previously termed Int3, which resulted in the constitutive expression of a cleaved form of Int3/NICD4, which drives the formation of mammary tumors [107, 108]. The expression of NOTCH1-signaling components is greatest in the luminal lineages, and forced expression of the active NICD1 in CD24^+^/CD29^hi^ mammary stem cell-enriched population directs this population towards the luminal cell lineage [106]. Conversely, knockdown of CBF-1, a target of NICD1 resulted in expansion and increased repopulating capacity of the mammary stem cell-enriched population. The effects of Notch in mammary stem cells are likely mediated at least in part via signals from Elf5 [87]. Interestingly, forced Notch signaling in luminal progenitor cells resulted in an expansion of this compartment and the formation of luminal (CK8^+^/CK18^+^/E-cadherin^+^/SMA^−^/p63^−^) hyperplastic alveolar nodules when transplanted into de-epithelialized mammary glands [106]. Similar regulation of human breast stem and progenitor cells is apparent in the human breast [109]. Further, inappropriate expression of Notch results in transformation of luminal progenitor cells. These data demonstrate that Notch is essential for restricting the expansion of mammary stem cells and for driving luminal cell commitment.

Recent evidence suggests a number of additional signaling pathways and transcription factors can regulate specific aspects of the mammary cellular specification [7, 9]. Hence, lineage specification and homeostasis of mammary stem and progenitor cells is governed by interactions among steroid hormone-signaling paths, lineage-specific transcription factors and signals derived from the extracellular niche.

## Stem cells in cancer

Substantial evidence confirms the existence of cancer stem cells in several tissues. The existence of cancer stem cells was demonstrated in human leukemia, where limiting dilution xenotransplantation of CD34^+^/CD38^−^ leukemic cells recapitulated the entire original tumor, whereas CD34^+^/CD38^+^ and CD34^−^ leukemic cells were not tumorigenic at any cellular concentration [110, 111]. Subsequent studies demonstrated that the tumorigenic potential was not restricted to these markers as human CD34^+^/CD38^+^ leukemia cells also produced tumors in mice [112–115]. In an attempt to isolate and understand tumorigenic from non-tumorigenic breast cancer cells, Al-Hajj and colleagues [40] used antibodies against CD24 (Heat stable antigen/HAS/BA-1) and CD44 (H-CAM/Pgp-1) to separate heterogeneous cell types in patient-derived xenografts grown in immune-compromised mice. These cell surface receptors bind cell adhesion molecules on neural stem cells and melanoma cells [116, 117]. Cells derived from leukocytes, endothelial cells, mesothelial cells, and fibroblasts were eliminated using the lineage markers CD2, CD3, CD10, CD16, CD18, CD31, CD64, and CD140b [40]. Lineage cells that were positive for CD44 and had no or low expression of CD24 (CD44^+^/CD24^−/lo^) were enriched for tumorigenic activity [40]. In contrast, CD24^+^ contained differentiated tumor cells that failed to form tumors. Further enrichment of tumorigenic cells was achieved with additional staining using an antibody to ESA or EpCAM. Importantly, ESA^+^/CD44^+^/CD24^−/lo^ cells isolated from patient xenografts in mice could give rise to the complete cellular heterogeneity observed in the primary tumor in serially passaged xenografts, hence lineage-/ESA^+^/CD44^+^/CD24^−/lo^ tumorigenic cells were self-renewing and multipotent cancer stem cells [40]. Whether these tumorigenic cells represent the cellular origins of breast cancers remains to be determined.

Since these experiments, an understanding of cancer stem cells has evolved rapidly [118–123]. The CD44^+^/CD24^lo^ definition has also been found to enrich for cancer stem cells in the Her2/Neu mammary tumor mouse model [124]. Similar to observations of cancer stem cell phenotypes in hematopoietic malignancies, CD44^+^/CD24^lo^ is not a universal marker of cancer stem cells in breast cancer. Furthermore, differences in the oncogenic mutations that drive transformation have profound effects on the frequency and phenotype of cancer stem cells. This is true for mouse models of mammary cancer. The CD24^+^/CD29^lo^/CD61^+^ immuno-phenotype enriched a population of cancer stem cells in Wnt1-driven tumors, and in part in tumors that develop in p53 heterozygote mice, but not in tumors driven by overexpression of Her2/Neu [125]. Although these studies clearly demonstrate the existence of small population of cancer stem cells, like the situation in normal mammary gland, no markers have been found to reliably define cancer stem cells in mammary cancers.

## The progenitor cell as the major origin of breast cancer

Stem cells could serve as the cell of origin of cancer as they are self-renewing, long-lived and hence likely to accumulate mutations over time [126–128]. Efforts have been made to match the intrinsic molecular subtypes of breast cancer with distinct cancer stem cells. Tumors could arise from committed progenitor cells, with enough residual multipotent potential to give rise tumor cellular heterogeneity, comprised of lineage-committed tumor cells and small proportions of tumor-initiating cells. Stem cells may acquire the first genetic aberration and give rise to progeny that is predisposed to further mutations, triggering the onset of the disease [119]. Tumors display a dichotomy of hormone-responsive and non-responsive cells, where expression of ER and PR is variable within each intrinsic molecular subtype [128]. This dichotomy resembles that observed in the normal mammary gland. Whether tumors arise from transformed mammary stem cells that acquire heterogeneity through clonal evolution, or whether cancer arises from committed progenitor(s) or transit-amplifying cells is still unclear. It is more likely that both mechanisms coexist with different incidence in distinct breast cancer subtypes.

Research in hematopoietic malignancies provides evidence that committed progenitors cells may serve as cells of origin for malignancy [129]. Similar results have been shown for breast cancers [52, 119, 130] as was demonstrated by Molyneux et al. in 2010 [131]. In this study deletion of Brca1 in Blg (β-lactoglobulin)-positive luminal progenitors from p53^+/−^ mice produced ER-basal tumors that phenocopied Brca1 mutant tumors, whereas depletion of Brca1 in the CK14^+^ basal progenitor produced basal-like breast cancers that did not resemble those found in human patients, suggesting that Brca1 tumors likely arise from a luminal progenitor. This work is supported by the result that the luminal population is larger in Brca1 mutation carriers and possesses growth factor-independent growth properties [52]. Similarly, constitutive activation of the Notch1 pathway in luminal progenitors resulted in their transformation and resulted in hyperplasias with luminal characteristics [106]. Transformed committed progenitor cells may also dedifferentiate and acquire stem cell-like properties. A model for committed progenitors that acquire stem cell properties during regeneration/wound-healing responses has being described for prostate testis and pancreas [51, 132, 133]. These data support the notion that malignancy may originate in the progenitor cell compartment and result in the establishment of heterogeneous subtypes of breast cancer.

Following this hypothesis, factors which determine the cell fate decisions of the normal progenitor cell are likely to be key determinants of tumor phenotype, response to therapy and molecular subtype. Thus, transcription factors that specify normal mammary cell fate, such as Elf5 or Gata3, also have a profound effect on tumor progression [64, 86, 89, 134, 135] and therapeutic resistance [86]. For example, forced expression of Elf5 in ER-positive luminal breast cancer cells reduced ER and FOXA1 expression and suppressed the luminal subtype  molecular signature [86]. Knockdown of Elf5 in basal breast cancer cells also resulted in a shift in their molecular signature toward a claudin-low and normal-like subtype of breast cancer suggesting that Elf5 is a key determinant of breast cancer intrinsic molecular subtype. Elf5 expression is greatly increased when MCF-7 breast cancer cells acquire Tamoxifen or Faslodex resistance by long-term exposure to these anti-estrogens, and these cells also become dependent on Elf5 for their proliferation, suggesting that Elf5 is a key determinant for anti-estrogen resistance [86].

## Additional sources of breast cancer heterogeneity

In addition to the cell of origin, many other factors contribute to the observed molecular tumor heterogeneity [136–139]. Transcription factors figure prominently in the differential signatures [64, 85, 86, 140, 141]. Tumors exhibit marked morphological heterogeneity [127, 142]. Clonal evolution of tumors could result in tumor heterogeneity [143] via the acquisition of advantageous mutations that confer survival advantages [126]. Furthermore, heterogeneity may arise in response to the tumor cell microenvironment, where for example, tumor cells directly in contact with blood vessels are different from those in an anaerobic environment far from the vasculature [144]. The effect of the microenvironment is a critical source of tumor heterogeneity [126, 145, 146]. The suggestion that mammary stem cells are located in a ‘suprabasal’ niche within the mammary gland [22, 32, 47, 48, 147], imply that such cells will be poised to respond to extracellular cues that regulate their fate [148]. There are examples in multiple tissues suggesting that the niche provides signals to maintain the self-renewal capacity of stem cells and upon exiting of this niche, stem cells undergo differentiation [149]. The existence of a stem cell niche [150], and the effect of hormones and paracrine factors on mammary stem cells during mammary gland development [88, 151–153], may similarly affect cancer stem cells resident in breast tumors. Furthermore, tumors contain more than tumor cells; for example, endothelial cells, immune-infiltrated populations and fibroblasts. Each cell type plays an important role during the initiation and progression of the disease [154–156]. Epigenetics have been shown to play a strong role during development [157] including mammary development [104, 158], defining different lineages and specifying epithelial functions. Therefore, it is reasonable to think, epigenetic remodeling is similarly crucial for the definition of tumor populations as a mechanism to define committed decisions [159–161]. Together with genomic instability, the underlying epigenetic mechanisms of normal mammary morphogenesis must be considered as a key factor that contributes to tumor heterogeneity [162] as many types of cancer retain certain a hierarchal organization. In mouse models of mammary cancer, distinct oncogenic insults produce different cancers supporting the importance of the genetic mutation for the generation of heterogeneous tumor phenotypes. [125, 163, 164].

## Implications for therapy

Cancer stem cells provide an attractive target for the development of novel cancer therapies and are likely arbiters of the failure of anticancer therapies and relapse [165]. Furthermore, cancer stem cells are considered to be intrinsically resistant to chemotherapy [162] and radiation [166]. Endocrine therapies, such as tamoxifen and aromatase inhibitors for ER^+^ disease or herceptin, have proven efficacious [167], but may only target the ER^+^ and HER2-overexpressing mature tumor cell types and not multipotent undifferentiated cancer stem cells [128]. These therapies may also target the tumor microenvironment and indirectly affect tumor cell interactions with the cancer stem cell [168]. For example, it has been demonstrated that a mechanism of action of trastuzumab is to attract immune cells to the tumor site generating antibody-dependent cellular cytotoxicity [169, 170].

New treatments have been developed that have been designed to target cancer stem cells. An 11-gene stem cell signature based on the Bmi-1 renewal pathway can predict poor prognosis in multiple types of cancer including breast cancer [171]. New therapies include those that induce lineage maturation of cancer stem cells. Such a therapy has been used in acute promyelocytic leukemia [172] or glioblastoma [173], producing growth arrest in tumor cells by forcing their terminal differentiation. The rational behind this type of therapy is not only to overcome intrinsic cancer stem cell resistance and sensitize them to conventional therapy, but also to prevent tumor cell dissemination. This is because it has been suggested that although differentiated tumor cells are able to seed in distant organs, only the cancer stem cell will produce clinically relevant macrometastasis [162, 174].

## Conclusions and Perspectives

**Fig. 1:**
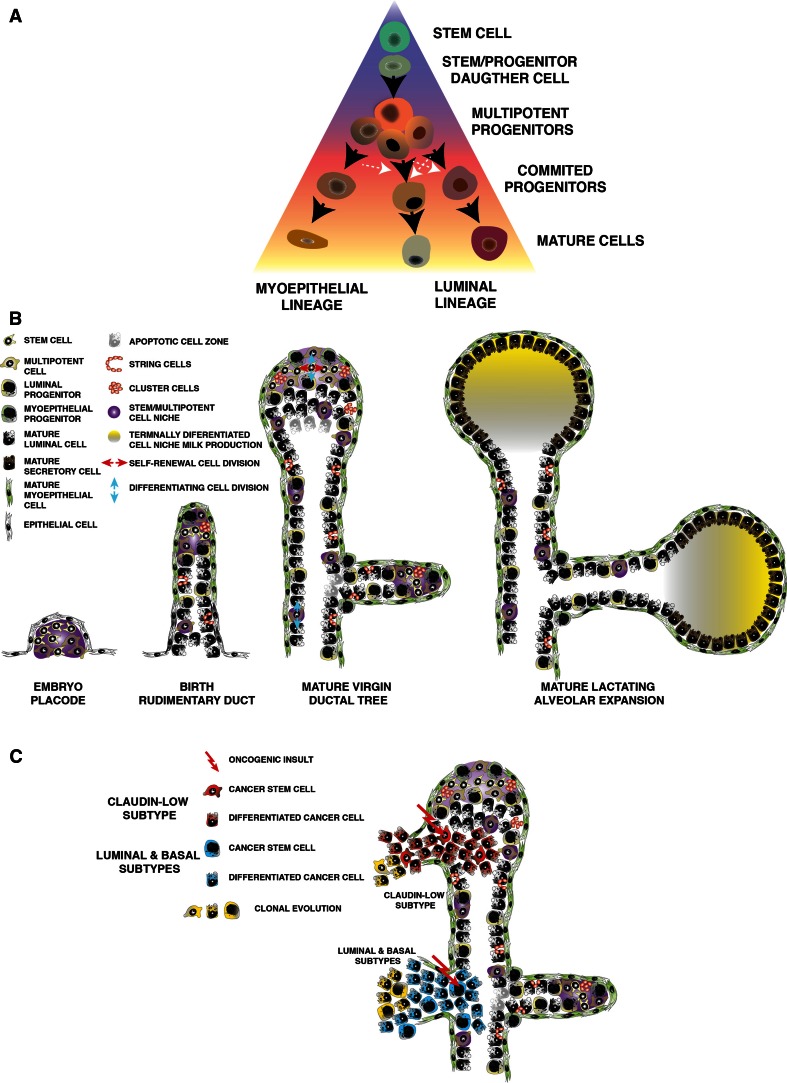
The stem cell niche during mammary development and carcinogenesis. **a** Traditional view of the mammary hierarchy: a mammary stem cell resides at the top of the hierarchy, present in the embryonic mammary gland and possibly in the adult. It gives rise to committed bipotent progenitor cells, which under the influence of extracellular and intracellular cues give rise to progenitors and mature cells in their respective lineages. **b** The mammary hierarchy exists within a cellular niche that has different activities, and locations, with each developmental stage. **c** The activity of the niche is disrupted during carcinogenesis, the exact nature of which may produce the heterogeneous cancer subtypes observed

The mammary cellular hierarchy exists as a dynamic state and which is intrinsically plastic (Fig. 1, cell types from the seminal publication of Chepko and Smith [20]). The multipotent stem cell is thought to sit within a controlling niche, where is poised to respond to exogenous cues and signals from the niche microenvironment, to give rise to more lineage-restricted oligopotent stem cells during post-natal development. We know very little about the nature of the niche, from its composition to the way it may control mammary stem cell function, and these aspects are among the key unanswered questions of mammary biology. We can infer a little more regarding its location. During embryogenesis, the mammary placodes form and invade the underlying mesenchyme, to form the rudimentary ductal network found at birth [176]. Within the placode, the initial niche architecture is presumably laid down, with the leading edge of invasion carrying a niche into the mesenchyme. During ductal elongation, the niche reproduces itself while also depositing copies along the length of the duct. Puberty activates the multipotent stem cells at the ductal termini, which may divide in specific directions, so that division along the plane of ductal elongation produces myoepithelial-committed oligopotent stem cells in the forward direction and luminal-committed oligopotent stem cells in the reverse direction. In turn, these stem cells produce the two major cell lineages of the mammary epithelium, with terminal end bud cap cells, derived from forward stem cell divisions, becoming the myoepithelial layer, and the terminal end bud body cells, derived from reverse divisions of the stem cell differentiating to become the luminal lineages. If the stem cells divide in a direction across the niche they may reproduce themselves, and so seed the mammary epithelium with multipotent stem cells as the terminal end bud advances. As ductal elongation proceeds, the niche bifurcates to produce the characteristic “Y”-shaped branch points. These myoepithelial and epithelial niches deposited along the duct must cooperate to produce ductal side branching, recognized as “T”-shaped branch points, in response to estrous/menstrual cycles and pregnancy [182, 183]. Alternatively, there may be only one type of niche and stem cell, which is able to vary the type of cells it produces depending on context. Transplantation, or the conditions of initial mammary development postpartum or at puberty cause it to act as a mutipotent stem cell in response to wound-healing stimuli, while lineage tracing in vivo shows it to behave as an oligopotent stem cell with more restricted epithelial or myoepithelial commitment [91, 92]. Non-Waddington stem/progenitor cell dedifferentiation and transdifferentiation should also be considered as a response to disruption of the niche. How the niche may contribute to cancer heterogeneity is also a key question. Currently, it appears that the luminal progenitor is the basis of most breast cancers, comprised of luminal and basal subtypes (Fig. 1) with the stem cells producing much rarer mesenchymal subtypes such as the claudin-low cancers. Additional progenitor lineages may yet be discovered, say for example one driven by progesterone that may account for the luminal B type of cancers [175]. If the stem cell is the target of oncogenic change, and the niche retains its responses to external stimuli, then a wide variety of tumor phenotypes could be produced from the same core group of cells, with the niche replicating itself within the tumor and its metastases. New technologies such as lineage tracing, single cell whole genome analysis and our rapidly evolving understanding of epigenetic control make this an exciting time in mammary biology.

## Figures and Tables

**Fig. 2:**
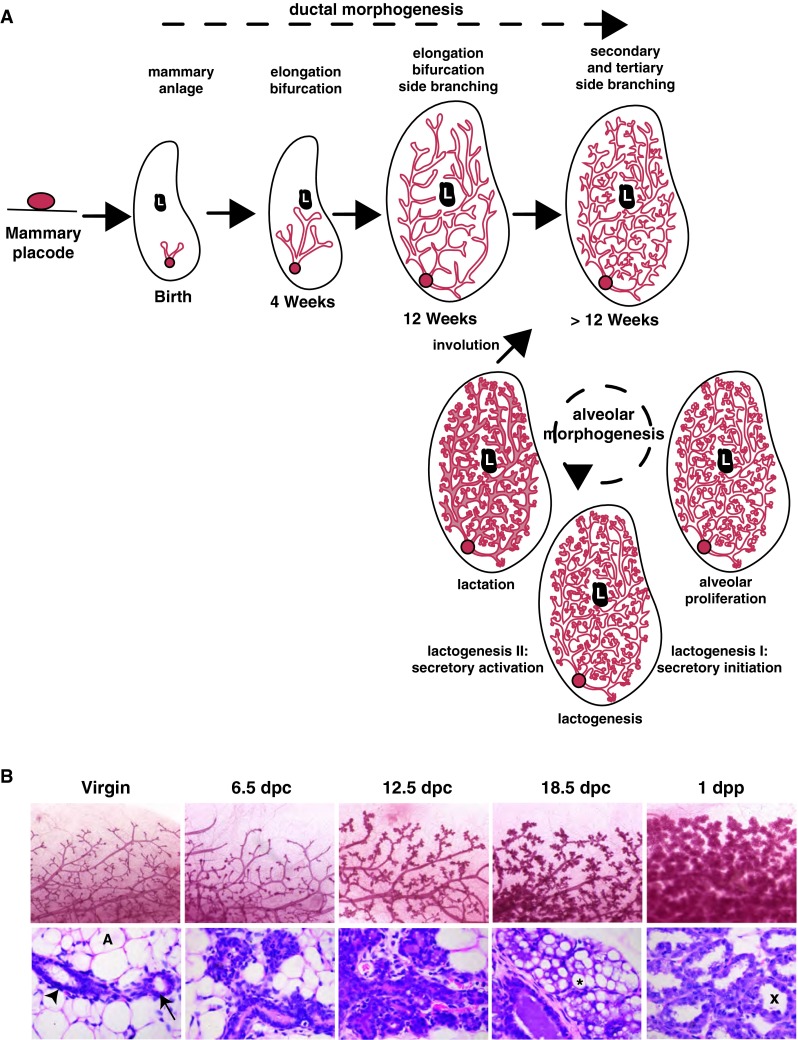
Mammary gland development, embryonic to pregnancy and beyond. **a** The mammary gland emerges from mammary placodes that form along the milk line at embryonic day 10–11. At birth, the mammary epithelium emerges from the placode and grows isometrically until puberty when rapid expansion of the epithelium extends the ducts throughout the fat pad. Ductal side branches sprout from the lateral surfaces of the ducts in multiple rounds of proliferation throughout each estrous cycle and results with the complete filling of the mammary fat pad. This process is collectively termed ductal morphogenesis. Pregnancy induces alveolar morphogenesis where proliferation establishes the alveolar epithelium, which then differentiates into milk-secreting alveoli at mid-pregnancy (lactogenesis I/secretory initiation). The final phase of differentiation results in milk and lipid movement into the alveolar lumens ready for lactation (Lactogenesis II/secretory activation). Milk ejection maintains lactation, and after weaning the mammary gland undergoes rapid programmed cell death (involution), returning the gland to a near-virgin state. **b** Mammary whole mounts (Carmine alum stain *top row*) and mammary cellular architecture (hematoxylin and eosin histology *bottom row*) in virgin, 6 days post coitus (dpc), 12 dpc, 18 dpc and 1-day postpartum (1dpp) murine mammary glands. Ductal epithelial cells (*arrow*), myoepithelial cells (*arrowhead*) and adipocytes (*A*) are indicated. Alveoli form a sphere-like single layer of epithelial cells enveloping a circular lumen (*X*) and contain cytoplasmic lipid droplets from 18 days post coitus, after secretory initiation (*asterisk*). At parturition, tight junctions between alveolar cells close and milk proteins and lipid are secreted into the alveolar lumen (*X*). An expansion of the vasculature (*open arrows*) and reduction in area is also apparent in the stroma

## Appendix: Mammary gland morphology and development

The mature mammary gland is composed of a bi-layered epithelium consisting of luminal cells that give rise to the milk-secreting alveoli during pregnancy, surrounded by contractile myoepithelial cells that contribute to milk ejection during lactation. These layers of epithelium are in close proximity to the extracellular matrix (ECM) and sit in a bed of stroma consisting of adipocytes and fibroblasts, and infiltrated with the blood and lymph vasculature. Macroscopically, mouse mammary epithelium forms a complex network of ducts terminating in alveolar buds connected to the nipple by a single canalized duct. In the human, terminal ductal lobular units cap the ends of the ducts and are made up ductules and alveolar buds. Reproductive hormones from the endocrine system, together with secreted factors from epithelial cells and the extracellular matrix, drive cell fate and functional differentiation within the mammary epithelium in a spatial and temporal manner during all stages of mammary development.

Mammary gland or breast morphogenesis occurs largely after birth from a rudimentary ductal tree that forms during embryogenesis (Fig. 2). Mammary embryonic development gland is initiated at embryonic day 10–11 in the mouse and at 4 weeks in the human [57, 176], where primordial mammary buds emerge from thickenings of the ectoderm (placodes) along the milk line that forms primitive mammary buds and at birth the rudimentary ductal tree [176, 177]. Each duct is capped with terminal end buds which are composed of an outer layer of undifferentiated cap cells, inner layers of highly proliferative luminal epithelial cells and an apoptotic plate behind the luminal cells resulting in ductal canalization [178]. The rudimentary mammary epithelium enlarges isometrically until puberty, largely under the influence of growth hormone and estrogen [179–181].

Pituitary gonadotropins trigger the onset of puberty [182] and mammary ductal branching morphogenesis in response to oscillating levels of estrogen and progesterone during the estrous cycle [183, 184]. The cyclical surges of ovarian hormones post-puberty result in continuous rounds of proliferation and incomplete apoptosis, and result in the establishment of a complex network of ducts that penetrate to the extremities of the fat pad [185–188].

Pregnancy triggers alveolar morphogenesis and is under systemic control of luteal progesterone, pituitary prolactin and later placental lactogen. The mammary epithelium undergoes rapid proliferation resulting in increased branching and the development of the alveolar epithelium, which terminally differentiates into the alveolar epithelium capable of milk secretion at mid-pregnancy [189, 190]. At weaning, this newly formed alveolar epithelium regresses via apoptosis leaving the ductal epithelium largely as it was prior to pregnancy [191], although it is now recognized that some parity-induced mammary epithelial cells remain [79, 82] concomitant with permanent changes gene and protein expression [192].

## Abbreviations

ABCG2: ATP-binding cassette sub-family G member 2
ALDH1: Aldehyde dehydrogenase 1
Blg: β-Lactoglobulin
Bmi-1: B lymphoma Mo-MLV insertion region 1 homolog
BRCP1: Breast cancer resistance protein 1
BrdU: 5-Bromo-2′-deoxyuridine
CALLA: Common lymphoblastic leukemia antigen
CD133: Cluster of differentiation 133/prominin-1
CD24: Cluster of differentiation 24/heat stable antigen
CD29: Cluster of differentiation 29/β1 Integrin
CD44: Cluster of differentiation 44/H-CAM
CD49b: Cluster of differentiation 49b/VLA 2
CD49f: Cluster of differentiation 49f/integrin alpha 6
CD61: Cluster of differentiation 61/integrin beta 3
CD90/Thy1: Cluster of differentiation 1/Thymocyte differentiation antigen 1
CK5/K5: Cytokeratin/keratin 5
CK8/K8: Cytokeratin/keratin 8
CK14/K14: Cytokeratin/keratin 14
CK18/K18: Cytokeratin/keratin 18
CYP24A1: 1,25-Dihydroxyvitamin D_3_ 24-hydroxylase
cKIT: Tyrosine–protein kinase kit
DNA: Deoxyribonucleic acid
ECM: Extra-cellular matrix
Elf5: E74-like factor 5
EMA: Epithelial membrane antigen
EpCAM: Epithelial cell adhesion molecule
ER: Estrogen receptor
ERRB3: Receptor tyrosine–protein kinase erbB-3
ERBB4: Receptor tyrosine–protein kinase erbB-4
Foxa1: Forkhead box protein A1
Fzd8: Frizzled family receptor 2
Gata3: Trans-acting T cell-specific transcription factor 3
GFP: Green fluorescent protein
HER2: Human epidermal growth factor receptor 2/Neu
hTERT: Human telomerase reverse transcriptase
ID4: Inhibitor of DNA binding 4
Jag2: Jagged 2
Lgr5: Leucine-rich repeat containing G protein-coupled receptor 5
MFGM: Milk fat globule membrane antigen
MMTV: Mouse mammary tumor virus
MUC1: Mucin 1
Myb: Myb proto-oncogene protein
NEP: Neutral endopeptidase
NICD: Notch intracellular cellular domain
Nrg1: Neuregulin heregulin-alpha
p63: Tumor protein p63
PR: Progesterone receptor
Prlr: Prolactin receptor
RankL: Receptor activator of nuclear factor kappa-B ligand
SCA-1: Stem cell antigen
SMA: Smooth muscle actin
SOX11: SRY (Sex-Determining Region Y)-Box 11
SP: Side population
Stat5A: Signal transducer and activator of transcription 5A
Tcf4: Transcription factor 4
Tgf-β: Transforming growth factor beta
Tnfsf11: Tumor necrosis factor ligand superfamily member 11
WNT: Proto-oncogene protein Wnt
X-Gal: 5-Bromo-4-chloro-3-indolyl-β-D-galactopyranoside
YFP: Yellow fluorescent protein
